# Supplementary material for: Are carbon emissions trading and green financial instruments synergistic? -Comprehensive quantitative research based on content analysis
Source: PLoS One. 2024 Mar 7;19(3):e0298601. doi: 10.1371/journal.pone.0298601 (PMC10919601; doi:10.1371/journal.pone.0298601)
Supplement: S1 Data — (ZIP) [file pone.0298601.s001.zip › Data/地级市Z_F工作报告.zip!/╣≤╓▌/╣≤╤⌠╩╨/└┤╫╘╣1⁄2╓┌║┼í╛┬φ┐╦╩2╛▌═°í┐_2005.docx]

各位代表：

现在，我代表市人民政府向大会作工作报告，请予审议，并请各位政协委员和其他列席人员提出意见。

一、2004年工作回顾

2004年，在省委、省政府和市委的正确领导下，全市广大干部群众以邓小平理论和“三个代表”重要思想为指导，认真贯彻党的十六大、十六届三中、四中全会精神，以科学的发展观总揽全局，以全面建设小康社会和建设大贵阳为目标，按照省委九届五次、六次全会的安排部署，解放思想，抢抓机遇，开拓创新，扎实工作，努力克服前进中的各种困难，圆满完成了市十一届人大三次会议确定的各项目标任务，国民经济继续保持持续、快速、健康发展的局面，呈现出速度与结构、质量、效益协调发展的态势。预计全市实现生产总值438.48亿元，比上年增长13.8%，其中，第一产业实现增加值32.42亿元，增长7.6%；第二产业实现增加值230.8亿元，增长15.5%；第三产业实现增加值175.26亿元，增长12.5%。市委、市政府年初确定的10件实事基本完成,各项社会事业都取得了新的成绩。

（一）固定资产投资规模稳步增长，投资结构进一步优化。

按照中央关于宏观调控的各项政策措施，我市从实际出发，区别对待，分类指导，有保有压，适时适度地采取一系列措施，保持了固定资产投资稳步增长的良好态势。预计全市全社会固定资产投资完成292.78亿元，比上年净增50.91亿元，增长21.1%。其中：基本建设投资完成127亿元，增长18.4%；更新改造投资完成59亿元，增长10%；房地产开发投资完成70亿元，增长14.4%，为拉动全市经济增长和调整经济结构作出了积极的贡献。

（二）工业生产稳步增长，经济效益明显提高，非公有制经济快速发展。

多年来的国企改革和结构调整的成绩开始进一步显现，全市工业生产保持了强劲增势，增长的速度和质量进一步提高，企业利润总额大幅度提高。全市规模以上工业企业完成增加值164.88亿元，比上年增长17.6%。全市规模以上工业企业盈亏相抵后利润总额达22.98亿元，增长150.91%；市属扭亏增盈考核工业企业盈亏相抵后利润总额达3548万元，增长10.53%。烟草制品业、有色金属冶炼及压延加工业、医药制造业、化学原料及化学制品业、电力生产和供应业、交通运输设备制造业、橡胶制品业、通信设备、计算机及其电子设备制造业等行业增加值大幅增加。工业产品产销衔接良好，工业产销率继续上升，全市规模以上工业产品产销率达96.1%。贵州轮胎股份有限公司的前进牌全钢子午胎获得“中国名牌”称号，结束了贵阳市没有中国名牌产品的历史。加快工业经济结构调整，大力推动循环经济建设，出台了《贵阳市建设循环经济生态城市条例》。做大磷化工、铝加工、现化中药、机电等支柱产业，加快发展高新技术产品，改造提升传统产业。新产品产值和出口交货值增势强劲，以微硬盘为代表的新产品增长较快，全市规模以上工业实现出口交货值为24.71亿元，增长1.3倍。国有企业改革稳步推进,积极参与贵州山花乳业有限公司、贵州瀑布啤酒（集团）公司、商业汽车场、贵阳标准件一厂、贵阳轻工输送设备厂等企业的改革改制工作,推动贵州轮胎股份有限公司国有股权转让工作。非公有制经济发展迅速，全市非公有制经济增加值比上年增长16%，占全市GDP的比重首次突破40%，对经济增长和社会就业等作出了积极贡献。

（三）农业结构进一步调整，农村经济全面发展。

认真贯彻中央1号文件精神，制定出台了《关于促进农民增加收入的实施意见》等一系列扶持农业发展、促进农民增收的政策，采取有力措施，确保对农民的直接补贴、良种补贴、农业税减免等落实到位。继续以农业产业化经营为主线，以农产品质量标准体系建设为突破口，加大无公害农产品基地和特色农业产业基地建设力度，大力发展绿色食品，不断发展壮大农产品加工龙头企业。全年完成粮食总产量59.65万吨，增长5.9%，油菜籽产量5.88万吨,增长7.7%，蔬菜总产量达86.88万吨,增长5.4%。推进优质烤烟基地建设，完成烤烟收购30万担。农业基础设施建设步伐进一步加快，完成县乡公路改造和贫困村公路路基改造工程，完成坡改梯1.27万亩和7740亩旱能灌、涝能排、方便耕作的高标准农田。扶贫攻坚力度加大，完成扶贫项目135个，脱贫人口1.13万人，解决农村人畜饮水7.9万人。经过四年的努力，共完成茅草房改造37562户116.12万平方米，使15万贫困农民告别了祖祖辈辈居住茅草房的历史，实现了市委、市政府提出“举全市之力，用3到5年的时间全面消灭农村茅草房，彻底改善广大贫困农户居住条件”的庄严承诺。农村富余劳动力转移工作取得新进展，全市完成农村劳动力转移2.95万人，劳务输出增加农民收入1.6亿元。

（四）社会消费品市场稳中趋旺，服务业继续保持平稳增长态势。

住房、通讯、汽车三大消费热点持续升温，连锁超市、物流配送、电子商务等新流通业成为拉动消费需求快速稳定增长的亮点。国际连锁超市的入筑，带来了新的理念和新的管理模式。全市消费市场呈逐季升温之势，第三产业对国民经济的贡献作用进一步增强。全市社会消费品零售总额完成175.52亿元，同比增长14.2%。旅游业呈现良好的发展势头，积极发展生态旅游、文化旅游、休闲旅游项目，共接待国内游客1162.7万人（次），接待海外游客3.6万人（次），实现旅游收入达48.6亿元。

（五）财政收入稳定快速增长，城乡人民生活进一步改善。

全年财政总收入实现110.73亿元，比上年增长20.88%，地方财政收入完成49.7亿元，增长23.07%。同时，支出结构进一步优化，确保了重点建设、社会保障、支农及科教等公共支出的需要。在工薪收入增长的拉动下，城市居民人均可支配收入继续保持稳定增长态势，城市居民人均可支配收入达8989元，增长12.6%；农民人均纯收入实现2809元，增长11.9%。就业再就业工作进一步加强，新增就业岗位4.15万个，解决城镇失业人员就业2.67万人，下岗失业人员再就业1.71万人，城镇登记失业率控制在4.1%以下。社会保障体系进一步完善,“三个确保”实现应保尽保。

（六）投资环境进一步优化，招商引资进出口业务势头良好。

深入实施“环境立市”战略，进一步提高对外开放和招商引资水平，着力打造投资成本最低、回报最快、办事效率最高、服务最好的环境优势。强化招商引资目标管理，把招商引资作为“一把手”工程抓好抓落实，修改和完善了《贵阳市招商引资目标责任考核及奖惩试行办法》及《实施细则》，为建立健全招商引资责任制和激励机制、保证年度目标的顺利实现提供了有力的保障。进一步明确全市招商引资目标区域、目标产业和发展重点，不断创新招商方式，强力推进我市招商引资工作。排名世界500强之首的零售业巨头沃尔玛入驻我市。全市共引进内资项目1364个，实际到位资金122.42亿元人民币，比上年增长35.64%。直接利用外资5237万美元，增长18%。外贸进出口增势强劲，完成外贸进出口总额12.05亿美元，增长41.5%。

（七）城市建设和管理进一步加强，城市化和城市文明程度不断提高。

坚持新区建设、旧城改造、小城镇建设“三轮驱动”，城市化水平不断提高。加强了城镇重点工程、小城镇和人居环境建设。新区建设，在完成金阳新区道路主骨架建设的基础上，将工作重心转移到人口聚集和产业支撑上来，微硬盘成功下线，一批相关上下游企业正在入驻基地。新区内住宅、医院、学校等配套项目的建设进展顺利，全面完成了市级行政中心的搬迁，极大地推动了金阳新区的发展。老城区改造，以完善城市功能为中心，继续抓好市区道路改建、停车场、污水处理厂等一批重点基础设施工程。“南明河三年变清”系列工程取得重大胜利，实现了“水变清、岸变绿、景变美”的目标，获得“中国人居环境范例奖”。继续实施“净化、亮化、美化”工程，拆除违章建筑26万平方米，提升了城市形象。完成了一批中日环保示范城市项目，重点污染源得到治理，清洁能源工程进一步深入，环境质量不断改善。小城镇建设全面加速，区（市、县）政府所在地和一批中心集镇的基础设施不断完善。城市绿化和森林建设工作取得新的成绩，被评为“全国绿化模范城市”和“国家森林城市”，通过省级园林城市检查验收。加大“卫生与秩序”整治力度，市容市貌和交通秩序得到明显改观。“文明林城”、“诚信贵阳”建设力度加快，城市管理水平全面提升。

（八）社会各项事业取得新发展，民主法制和政府自身建设得到加强。

组织实施科技项目127项，评定科学技术奖45项。电子信息、新材料、机电一体化等高新技术产业集群初步形成，科技示范园、知识经济产业园区发展步伐加快。全面完成科技人才强市“百千万”培训工程，建成科技大厦。高新技术产业、科技示范园、知识经济产业园区发展步伐加快，知识经济产业化基地被命名为“科技部863产业化基地”积极实施企业信息化“1050”工程，全面推进“数字贵阳”建设。继续巩固和提高“两基”达标成果，修文、息烽通过“两基”达标复查验收，稳步推进基础教育课程改革、中小学布局调整和中小学危房改造，巩固普及九年制义务教育成果。整合教育资源，贵阳学院正式招生。首次发布全市知识产权白皮书，知识产权保护工作不断加强。加大了对县乡卫生基础设施的投入力度，进一步健全了市、县、乡、村四级疾病预防控制机构，扩大农村合作医疗试点。进一步加强计划生育工作，人口自然增长率控制在7‰以内。

民主法制建设不断加强。认真执行市人大及其常委会的决议，向市人大及其常委会报告工作并接受监督。支持政协以及各民主党派、工商联履行政治协商、民主监督和参政议政职能，及时通报情况并听取意见和建议。同时，发挥专家咨询作用，加强民情民意调查网络建设，完善听证制度，促进了政府决策的民主化和科学化。全年共办复省、市人大代表建议217件，办复省、市政协委员提案366件，办结率为100%。起草地方性法规草案11件，发布政府规章12件。行政执法监督机制逐步完善，依法行政工作得到加强。围绕创建“平安贵阳”，以构建打防控一体化社会治安长效管理机制为主线，不断深化严打整治斗争，有力地打击了刑事犯罪分子的嚣张气焰，社会秩序保持稳定，社会治安总体水平明显提高，人民群众安全感日益增强。积极推行政务公开，现行文件查询向市民开放。行政审批制度改革进一步深化，共减少市政府规章自行设定的行政许可事项77项。坚持标本兼治、综合治理的方针，加大从源头上预防和治理腐败的力度，严肃查处了一批违纪违法案件，反腐败斗争取得新进展。与此同时，统计、审计、物价、体育、广播电视、房管、外事侨务、民族宗教、对台、人防、档案、机关事务、民兵预备役、残联等部门工作取得了新的成绩。

各位代表，过去的一年，遇到的困难超出预料，经受的考验多年未有，取得的成绩来之不易。这是省委、省政府、市委正确领导和市人大、市政协有效监督的结果；是全市人民树立科学发展观，努力克服各种不利因素的影响，顽强拼搏、共同奋斗的结果；也是市委、市政府贯彻中央和省的方针政策、加大改革开放力度、推进经济结构调整的结果。在此，我代表市人民政府，向全市广大工人、农民、知识分子及其他社会主义建设者，向各位人大代表、政协委员，各民主党派、工商联、群众团体，向人民解放军、武警驻筑部队、预备役部队及中央、省属驻筑单位表示衷心的感谢!

在充分肯定成绩的同时,我们也清醒地看到，贵阳市目前面临着“内外夹击”和“前有标兵，后有追兵”的严峻形势，作为省会城市的贵阳，只有进一步加快发展，才能在全省经济发展中发挥龙头作用，作出应有的贡献。从2004年经济运行情况看，我市经济社会发展还存在不少突出的问题，主要表现在：制约经济发展的体制性障碍和结构性矛盾仍然突出；财政收支矛盾突出，调控能力弱；就业再就业形势严峻，部分下岗失业人员、城镇低收入居民和贫困地区群众的生活还比较困难；煤电油运供求紧张的矛盾尚未得到有效缓解，流动资金吃紧，信贷结构性矛盾比较突出；经济增长的基础还不够稳固，城乡差距较大，“三农”问题依然突出等等。对于这些问题，我们将高度重视，着力研究，认真加以解决。

二、2005年经济社会发展的主要任务

今年是贯彻落实科学发展观，加快建设生态经济市、为实现建设大贵阳目标努力奋斗的关键一年，也是胜利完成“十五”计划、全面衔接“十一五”规划的关键一年。贯彻中央和全省经济工作会议精神，结合我市实际，今年工作的总体要求是：以邓小平理论和“三个代表”重要思想为指导，认真贯彻党的十六大和十六届三中、四中全会和省委九届六次全会精神，认真贯彻中央经济工作会议和全省经济工作会议精神，更加坚定不移地贯彻发展是硬道理，更加坚定不移地贯彻发展这个党执政兴国的第一要务，更加坚定不移地树立和落实科学发展观，用科学发展观统领经济社会发展全局，进一步提高驾驭社会主义市场经济的能力，围绕全面完成“十五”计划、科学谋划“十一五”规划，切实贯彻落实宏观调控措施，以推进经济结构调整、转变经济增长方式为主线，以改革开放和科技进步为动力，加快城市现代化、农村城镇化、工业新型化、农业产业化步伐，正确处理改革、发展、稳定的关系，努力提高人民生活水平，实现经济社会更快更好地发展。

今年经济和社会发展的预期目标是：全市生产总值增长13%左右。其中：第一产业增加值增长7%，第二产业增加值增长14.5%，第三产业增加值增长12.5%。固定资产投资比上年净增50亿元；地方财政收入增长12%；社会消费品零售总额增长13%；进出口总额增长15%；实际利用外资增长10%；非公有制经济增长16%；城镇居民人均可支配收入增长8%；农民人均纯收入增长8%；城镇登记失业率控制在4.6%以内；人口自然增长率控制在7‰以内。全面完成和超额完成“十五”计划。

市委、市政府为民办好的十件实事是：

1、关心困难群众：（1）新增就业岗位3.8万个；（2）完成“双困户”廉租住房安置800户；（3）建成两城区低保群众“爱心超市”16个；（4）建立农村独生子女户、双女结扎户养老保障制度和女职工生育保险制度；（5）免费对贫困农民儿童实行计划免疫，免费对传染性肺结核患者实行全程治疗；（6）享受最低生活保障标准的城市居民免收市属医院就医挂号费和诊察费，除药费以外的其它费用减免10%。

2、加强便民服务：（1）建成贵阳政务服务中心并投入使用；（2）建成10个“三农”科技专家服务中心；（3）办理历年来市政基础设施安置房产权证2000套，完成房改房市场准入证审核办理20000户；（4）构筑农村维权网络“服务联络点；（5）免费为社会公众提供文件、档案信息资源服务。

3、改善农村生活环境：（1）建设100个经济生态村；（2）完成“四改一气”农村生态建设项目20000户；（3）完成农村5.7万人畜饮水及万户小水池工程；（4）完成二环林带建设5.39万亩，进行市级退耕还林工程试点。

4、加强农村基础建设：（1）完成500公里县乡公路建设改造工程和1000公里贫困村村公路建设改造工程；（2）完成9处铁路道口“平改立”工程；（3）建设100个农村党员活动室和80个贫困村宣传文化室；（4）完成447个行政村“模改数”工程和800个自然村通广播电视工程。

5、完善城市基础设施建设：（1）在金阳新区建设2条城市道路；（2）在市中心区建设7条城市道路，建设3座人行过街设施；（3）新建15座公厕、8个垃圾转运站、升级改造4个垃圾转运站，新建3个集贸市场；（4）贵阳大剧院工程竣工并投入使用；（5）建成贵阳市消防指挥中心；（6）建设雅关截污沟。

6、继续改善城市人居环境：（1）新增2500个公共停车位；（2）完成两城区3个住宅小区环境综合治理；（3）在贵阳市城区公交车上开通移动电视；（4）新增30万平方米绿地，创建10个绿色社区；（5）经济适用房开工60万平方米，竣工40万平方米。

7、改善教学条件：（1）继续加强市直属薄弱学校建设，重点改善4所薄弱学校的办学条件；（2）实施20个农村中小学危房改造项目，消除农村中小学现有危房1万平方米；（3）建设农村中小学小伙房、厕所各630个；（4）在城郊结合部流动人口聚居地改扩建公办学校4所，接纳更多进城务工农民子女就读。

8、建立健康医疗体系:（1）构建贵阳市国民体质监测体系，建成市国民体质监测中心和6个区国民体质监测站;（2）建成贵阳市紧急救援中心并投入使用;（3）改扩建贵阳市传染病医院;（4）建成7个区、市、县传染病区并投入使用。

9、加强公共安全体系建设:（1）完成两城区20个社区居民生活、医疗卫生、安全防范呼叫信息服务系统;（2）加强农村药品供应网络和药品监督网络建设，方便和保障农民安全用药。

10、加强法治基础设施建设：（1）完成20个乡镇司法所办公用房建设；（2）改造建设7个区、市、县人民法庭。

同时，今年还将做好“十一五”规划的制定工作。坚持以科学发展观为统领，把以人为本的施政理念和五个统筹的要求贯穿于规划的各个方面，实现好、维护好广大人民群众的根本利益。

为实现上述目标任务，更快更好地发展贵阳经济，做大做强贵阳市，实现经济社会跨越式发展，今年我们将重点抓好以下工作。

（一）大力推进结构调整，促进经济快速增长

一是继续推进“工业强市”战略。坚持以市场为导向，以技术创新为动力，依托资源优势，节约资源，注重环保，优化提升磷化工、铝及铝加工、煤化工、装备制造、卷烟、能源等现实优势产业，大力发展现代中药、特色食品等具有竞争优势的行业，优先发展电子信息、新材料、光机电一体化等高新技术产业和产品。加快安大、高峰、大唐高鸿、EADS、法国斯莱克玛、TDK等航空、航天核心项目的引进和建设进度，力争年内形成现实生产力。完成贵州轮胎股份有限公司200万条半钢子午线轮胎生产线建设。重点抓好南方汇通微硬盘、世华微硬盘、表面贴装分立元器件（SMP）后封装生产线、高压真空灭弧室生产线技术改造、通过GMP认证的制药企业等一批达产增效项目。尽快建成投产中化开磷集团磷酸二铵生产线、硫基复合肥生产线、苏西铜项目、中铝氧化铝节能、华光铝型材二期、西南工具（集团）有限公司空调压缩机叶片技术攻关与产业化、贵航汽车零部件产业化等一批在建技改项目。抓紧实施威门药业地道苗药头花蓼、民族药业大果木姜子栽培及制剂、贵阳同济堂药业淫羊霍中药材保护抚育及提取加工、新材料矿业公司耐高温多孔泡沫特种陶瓷等一批国家高技术产业化项目。进一步发挥好生产力促进中心和风险投资公司的作用，为中小企业创建更好的服务平台，提升中小企业的科技含量，促进企业的科技进步。

二是加快培育新兴产业和高新技术产业。依托开发区、工业园区和特色产业基地建设，进行经济结构战略性调整。充分利用贵阳的资源优势，大力发展污染小、能耗低、效益好的电子信息产品制造、新材料、现代中药和特色食品、环保产业等新型产业和高科技新兴产业。充分发挥三个开发区特别是高新技术产业开发区的优势，围绕以微硬盘为中心的所有项目，搞好招商引资和综合服务工作，使其配置更加优化，效益更加明显,实现在高新技术方面的新突破。同时，进一步采用高新技术和先进适用技术，改造和提升传统产业，努力造就一批拥有自主知识产权的高新技术企业。加强政策和金融支撑，扶持高新技术产业加快发展，创造良好的企业成长环境。

三是加快发展现代服务业。加快发展服务业，提升我市的竞争力,在指导思想上满足不同层次人员的消费需求，优化政策措施，鼓励境外资本、民间资本、国有资本投资我市现代服务业。进一步发展金融业，积极引进和培育股份制银行，引导、规范、有序发展租赁公司、担保公司和风险投资公司。拓宽三产新领域,改造提升交通运输、商贸流通、餐饮等传统服务业，积极发展房地产、现代金融保险、物业管理、旅游、社区服务、信息服务、中介服务等新兴服务业，提高服务水平和技术含量，促进服务业向社会化、规模化、现代化方向发展,促进服务业发展的新跨越。紧紧围绕打造西南地区商贸中心的目标，大力发展物流配送、物流园区和大型批发市场，推进二戈寨物流园区、阳关物流园区、客流园区枢纽总站、浙江商城、金阳商贸走廊等项目建设。积极开发红色旅游、生态旅游和民族风情旅游，做好旅游产品的整体规划和促销工作，带动旅游业加快发展。

四是大力发展非公有制经济。认真贯彻落实《国务院关于鼓励支持和引导非公有制经济发展的若干意见》，大力优化各种所有制经济共同发展的政策环境、政务环境、法制环境、市场环境和舆论环境，力求在切实解决非公有制经济的平等待遇上有新的突破；在提高政府服务效率、建立非公有制经济发展协调机制上有新的突破；在加大对损害非公有制经济发展环境的责任追究上有新的突破；在建设特色园区、促进产业和要素聚集上有新的突破，促进一切生产要素的活力竞相迸发，一切创造财富的源泉充分涌流。进一步放宽非公有制经济进入基础设施、垄断行业、公用事业以及法律法规未禁止的其他行业和领域，重点引导非公有制经济发展农产品和资源深加工产业、劳动密集型产业、高新技术产业和服务业。加强非公有制经济的服务体系建设，包括政策、融资、咨询、行政等服务都要配套衔接，切实为非公有制经济发展解决实际问题。

五是做强做大县域经济，促进各地经济社会协调发展。继续加强经济强县建设工作，把培育壮大县域经济作为增强全市综合实力、统筹城乡发展的重大战略举措来抓。以新型工业化为核心，以结构调整为主线，以扩大开放为主战略，不断优化区县经济的结构、壮大区县经济的总量。立足区县各自比较优势，培育特色经济。加快发展工业小区和工业小城镇，促进产业聚集，大力提高工业化水平。加快特色农产品基地建设和龙头企业的培育，塑造品牌优势，发挥规模效应，进一步提高农业产业化水平。加快特色市场、特色区街的建设，形成一批各具特色而又极具影响力的物流集散地，努力提高服务业水平。本着“宜农则农，宜工则工，宜商则商”原则，放手发展民营经济。鼓励民营经济参与国有、集体包括乡镇企业的重组改造，引导民间资本投资兴办工业、城市公用事业和农村基础设施建设，使民营资本的活力得以充分释放和迸发。壮大区街经济，围绕增加就业，大力发展商贸流通、家政服务、医疗卫生、文体娱乐、物业管理、托幼托老、服装加工等社区服务业，发展现代物流、中介服务、城市旅游等新兴产业，不断增强县域经济实力，实现农民增收、企业增效、财政增长，促进城乡经济社会协调发展。

（二）着力抓好投资、消费和进出口工作，拉动经济快速增长

一是以科学的发展观进一步优化投资结构，提高投资效益。按照国家和省扶持的重点，高质量地准备基础产业项目、优势产业项目和关系群众切身利益的公益性项目，争取更多的项目列入国家和省的建设规划。抓好重点项目建设，围绕贵阳机场改扩建、贵阳铁路枢纽建设、贵遵路扎佐至南白段改造、修建贵阳过境高速公路等重大项目，抓好协调服务，努力为项目建设创造良好的外部环境，促进这些项目尽早开工建成，充分发挥重点建设项目对投资增长和经济发展的支撑作用。着力抓好“三个一批”。即认真清理一批，按照国家要求，对技术含量低、明显超出市场需求、不符合结构调整要求的项目，特别是盲目投资和低水平扩张的高能耗、高耗水、高物耗及污染严重的项目予以清理；重点支持一批，对技术含量高、符合结构调整要求的项目、缓解瓶颈制约的交通建设项目，以及需要加强的农林水利、生态建设、环境保护、社会事业等薄弱领域的项目给予重点支持。对国债支持领域的项目要多方跑办，努力争取国家和省支持；积极谋划一批，对有利于结构调整的技术改造、结构升级等项目，尤其是我市主导产业的项目，扎扎实实做好前期工作，不因为当前的调控政策延误工作的推进。与此同时，按照“投产一批、续建一批、新开工一批、储备一批”的要求，抓好项目库建设。放宽社会资本的投资领域，鼓励社会资本进入法律法规未禁入的基础设施、公用事业及其他行业和领域。利用特许经营、投资补助、贷款贴息、税收优惠等措施，鼓励和引导社会资本以独资、合资、合作、联营、项目融资等方式，参与经营性的公益事业、基础设施建设，激活各类投资主体，全面促进社会投资，努力扩大投资规模，确保实现固定资产投资比上年净增50亿元的目标。

二是抓住提高居民购买力和完善市场体系两个关键环节，促进消费优化升级，推动经济增长。完善消费政策、改善消费环境，培育和扩大消费热点，积极促进消费增长。高度重视在经济发展中增加人民群众的收入，调节好收入分配关系，保护好弱势群体利益，尽可能地提高人民群众的现实购买力。进一步加强各要素市场、中介市场、劳动力市场、批发市场、旅游市场等市场体系建设，引进更多的商业服务企业，真正做到货畅其流，为促进各方面消费的发展提供良好的市场体系，更好地发挥中心城市辐射带动作用。

三是进一步扩大我市的进出口，增强出口对经济增长的拉动力。进一步落实国家有关进出口的规定、政策和要求，尽快适应国家政策的调整，巩固和扩大出口。发挥好口岸协调领导小组的作用，加大对出口企业的服务力度，切实帮助他们解决困难和问题。积极争取国家中小企业国际市场开拓资金，用好贵阳市外贸发展资金，支持和鼓励企业走出去。加大对化工、绿色食品、五金机电、高技术产品出口的支持力度，形成具有地方特色的出口格局。用好出口奖励政策，兑现承诺和奖励，鼓励企业通过出口来做大做强，把以质取胜和多元化的出口战略落到实处。

（三）统筹城乡协调发展，开创“三农”工作新局面

一是紧紧抓住农民增收这个核心，抓好各项促进农民增收政策的落实。深入开展去年和今年两个中央1号文件和市委〔2004〕9号文件等一系列支农政策的宣传，真正让广大农民了解政策、理解政策、掌握政策。抓好各项支农政策和支农资金的兑现，落实好农业税减免、粮食直补、良种补贴和农机补贴等政策，让农民从中得到实实在在的好处。

二是优化产业布局，积极推进结构调整。坚持因地制宜和可持续发展的原则，充分发挥区位优势，在高等级公路、国道和省道沿线建设各具特色的商品化生产基地，形成一批各具特色的农业产业带。以提高农产品质量和效益为重点，加快优势农产品产业带建设，壮大和提升优质专用无公害蔬菜、奶牛、禽蛋、瘦肉型猪农业特色主导产业。全力扶持发展龙头企业、专业批发市场和农村专业合作组织，加快形成产供销、贸工农一体化的经营格局。做大做强农副产品加工业，重点扶持一批覆盖面广，带动能力强，能促使农民增收显著的龙头企业，以此带动其他相关产业的发展。

三是加强农业农村基础设施建设。继续坚持“水、电、路、气、房、林、电视、电话”十字方针，加大农村硬件基础设施建设的力度，改善农民生产生活条件，提高农业综合生产能力和抵御自然灾害的能力。继续实施县乡公路改造和贫困村公路路基改造工程。实施“四改一气一种一养”工程，完成中低产田改造、标准农田建设和坡改梯5.9万亩，改造危房3000户，完成农村5.7万人畜饮水及万户小水池工程，建设100个生态经济村。

四是加快转移农村剩余劳动力。建立健全劳动市场信息网络和劳务输出组织体系，落实培训资金，进一步搞好职业技能培训和就业指导，尽快提高我市劳务输出的竞争力，促进农村剩余劳动力有序转移，全年新增劳务输出2万人。全面清理和取消对农民工的歧视性政策和各种乱收费，完善农民工工资保障制度，加大对拖欠农民工工资的检查和处罚力度，维护进城务工人员的合法权益。

五是加大扶贫攻坚力度。切实把扶贫开发作为做好“三农”工作的重中之重，进一步加强分类指导，整体推进，突出扶贫工作的针对性，集中解决好帮助贫困群众改善基本生产生活条件、拓宽基本增收门路、提高基本素质这三个问题，帮助更多的贫困群众脱贫致富。进一步扩大农村社会救助制度覆盖面，有步骤地解决好鳏寡孤独、因病因残丧失劳动能力的特殊贫困人口的生活保障问题。同时，加强扶贫项目和资金的审计监督，提高资金使用效益。进一步加快农村小康建设步伐，使更多群众过上小康生活。

（四）着力推进经济体制改革，全面提高对外开放水平

一是继续深化国有企业改革。以国有企业改革“新三年目标”为重点，推进国企改革，调整国有经济的布局和结构，完善国有资产管理和监督体制，更广泛地推行以投资主体多元化为特征的股份制，实现政府公共管理职能和国有资产出资人职能分开。按照建立现代企业制度的要求，进一步完善企业公司法人治理结构、财务制度和决策机制。完善企业领导人的聘任制度，推行企业经营者年薪制。抓好缩小债务工作和缩小债务后的企业重组工作，利用多种手段，使更多的企业得到重组，产生更好的效果。

二是加强国资体制改革。进一步理顺所有权、使用权、经营权既统一又分离的关系，尤其要理顺国资委、国资公司、控股公司的关系，使整个体制更加协调，更加充满生机和活力，为全市的经济发展和社会进步提供良好的体制支撑和保障。

三是强化投融资体制改革。确立企业的投资主体地位，对于企业不使用政府投资建设的项目，一律不再实行审批制，区别不同情况实行核准制和备案制。全面推行“代建制”，建立投资决策责任制，实行政府投资项目公示制度和重大投资项目后评估制度。尽快适应国家推行核准制和备案制改革所带来的变化，改革和完善投融资政策、产业扶持政策，以及与投资经营活动相关的土地、税收、劳动就业、社会保障等政策，促进投资多元化、社会化，产业政策明晰化、公开化，产权操作公开化、市场化。充分发挥市场配置资源的基础性作用，营造有利于各类投资主体公平、有序竞争的市场环境，促进生产要素的合理流动和有效配置。充分利用银行、证券、资本市场等进行融资，协调好与各大银行的关系，发挥好市商业银行的作用，有效地提高我市的融资水平。进一步落实项目法人制、招投标制、工程监理制和审计验收决算制，加强管理，堵塞漏洞，提高效率，防止腐败。

四是继续深化农村改革。巩固税费改革成果，认真落实好全部免除农业税的政策，切实减轻农民负担。贯彻落实《国务院关于深化改革严格土地管理的决定》，坚持农村基本经营制度，规范农村土地流转，依法保障农民的土地承包权、生产自主权和经营收益权。积极稳妥地推进农村信用社改革，加大粮食购销企业产权制度改革力度。

五是进一步深化市政公用事业的体制改革。加快公交总公司、自来水公司、煤气气源厂、新建污水处理厂等一系列改革。按照特许经营管理办法，实现投资主体多元化、运行主体股份化、公益事业市场化、服务水平大众化、服务质量标准化。

六是深化政府行政体制改革。深入贯彻《行政许可法》，减少审批项目和审批程序，发挥好政府调控和市场调节的作用。贯彻国务院《全面推进依法行政实施纲要》，积极推进政府法制建设，努力创造依法治市的良好环境。加强服务型政府的建设，建立新的“政务服务中心”，为投资者和市民提供公开、公平、公正和高效率的服务。加强政府的效能建设，进一步提高行政效能和服务水平，打造为民、清廉、高效的政府。加强政府的社会管理职能，提高政府构建和谐社会的能力。

七是全方位扩大对外开放。进一步抓好软环境建设，以良好的法制、信用、服务环境来加大招商引资的力度，吸引更多的市外、省外、国外资金参与我市优势产业项目的建设和重组。以完善招商引资的工作方式和考核奖励机制为重点，进一步调动各方面招商引资的积极性、主动性和创造性，不断创新招商引资方式，做好小团队、专业化、针对性及网上招商等等。加强政务服务中心建设和三个开发区的对外开放工作，形成各有侧重、各扬优势、各尽其力、各有突破的相互竞争和合作的机制，最大限度地提升资源整合和投资优化的水平，把政务服务中心和三个开发区建设成为对外开放的重要窗口。积极参加泛珠江三角洲、南贵昆经济区、西南地区城市政府联席会，扩大与省内外城市的经济合作和技术交流。主动承接发达地区的产业转移，实施东西广泛合作。加强对外经贸合作，大力发展开放型经济。

（五）加快生态经济市建设步伐，切实转变经济增长方式

一是加快生态经济市建设步伐。按照市委、市政府出台的关于建设生态经济市的决定，认真做好城市生态经济总体发展规划，搞好宏观战略部署，促进城市生态经济整体优化，生态与经济实现良性循环，确保在经济持续快速发展的同时获得生态效益和生态功利的最大化。

二是大力发展循环经济。认真贯彻落实《贵阳市建设循环经济生态城市条例》，组织实施好《贵阳市建设循环经济生态城市总体规划》，落实第一批总投资248亿的24个项目，为启动项目的大企业集团搞好配套服务，制定鼓励发展循环经济的政策措施，逐步建立和完善促进循环经济的机制和体系。大力推进开阳磷煤化工生态工业示范基地、清镇煤化工基地、息烽磷复合肥基地建设。继续抓好循环经济试点工作，巩固试点成果，扩大试点范围，将试点逐渐向开发区、工业园区、区域经济延伸，使我市发展循环经济的工作走在全国前列。

三是加强环境保护工作。坚持工业污染防治与城市环境保护、农村环境保护与城镇综合整治、生态建设与生态保护相结合，关闭浪费资源、污染严重的企业，淘汰落后生产工艺和设备，推行清洁生产。用高新技术改造传统产业，通过科技进步和转变经济增长方式消减污染。从源头上加强农业生产环境和投入品管理，全面推进农村环境保护工作。

四是全面推进生态建设，打造“林城”品牌。巩固“南明河三年变清”成果，大力实施“绿色”、“蓝天”、“碧水”工程，搞好城市生态建设和景观建设，按照创建国家园林城市目标定位，扎实开展创建国家园林城市工作，把贵阳建设成为最适宜生活居住的城市。

（六）继续推进“三轮驱动”的城市发展战略，提升城市规划建设管理水平

一是强化规划的龙头作用。按照建设大贵阳发展战略目标，开展城市近期建设规划编制，突出编制好中心城区、新城区、旧城改造规划和建成区重点地段控制性规划，充分发挥规划对城市发展的引导和调控作用，强化规划管理，严格执行规划审批制度，加强对规划实施的全过程监督，切实维护规划的权威性。

二是加快旧城改造步伐。坚持以人为本，完善功能，创造特色，打造精品和魅力贵阳。着重抓好基础设施建设、城中村改造、南明河长治久清和提高城市管理水平四件大事。加快“城中村”改造步伐，逐步消除城市中心区和城郊结合部的危旧房。加大力度规划建设一批污水处理厂、垃圾填埋场、停车场和市场，完善城市功能。同时，要突出抓好次干道改造、城郊结合部破损道路改造，统筹抓好亮化、绿化、美化、净化等各项工作，提高城市品位。

三是强力推进金阳新区开发建设。着重抓好17平方公里基础设施建设和社会服务体系建设,完善新区功能。加大房地产开发力度和配套设施建设，加快聚集人口。大力发展高科技产业、现代服务业，抓好以微硬盘为龙头的高新技术产业群,尽快培育支柱产业，扩大产业规模。建立高效管理体制，在探索和运用新的管理机制上有较大突破。建成和完善医院、学校、体育等一批功能性设施。

四是加快以县城、重点镇为重点的小城镇建设。坚持以交通干线为基础，以经济流向为主导，发展专业化和特色化的小城镇，逐步形成各具特色的卫星城镇、中心城镇和一般城镇协调发展的城镇体系。

五是加大城市资本经营的力度。加快市政公用事业市场化步伐，建立和完善政府特许经营制度和公开招投标制度，调动外资和社会资金投资城市建设的积极性，吸引外资、内资、民间资金参与供水、供热、污水处理、垃圾处理的建设和经营，选择单位和个体参与市政设施、园林绿化、环境卫生的作业和养护。

六是严格执行耕地保护制度。在用地方面做到有保有压，控制增量，盘活存量，对经营性土地一律实行招标、拍卖、挂牌出让。进一步搞好土地储备工作，增强政府调控土地一级市场的能力，加大查处土地违法案件的力度，加强土地的批后管理，依法收回违规出让和闲置的土地。

七是切实加强城市管理。进一步理顺城市管理体制，继续推动管理重心向区、街下移，充分发挥社区在城市管理中的基础性作用，形成权责一致的城市长效管理机制。重点搞好环境卫生、交通秩序、空气污染等综合整治工作，改善城市形象，提高城市品位，争创全国卫生城市和文明城市，努力争取获得联合国人居环境奖。

（七）统筹经济社会发展，促进各项社会事业全面进步

一是继续深入实施科教兴市战略。不断深化科技体制改革，加快科技基础条件平台、科技孵化器和投融资等中介服务体系建设，抓好新医药开发和中药现代化、信息化改造传统产业、新技术新产品研发、特色农业等五大科技专项的组织实施，加强知识产权的科学管理，提高科技创新和成果转化能力。大力发展教育事业。落实好市委、市政府即将出台的《关于加快教育改革和发展的意见》，全面推进素质教育，提高教育质量和办学水平。继续高质量、高水平普及九年义务教育，落实好“以县为主”的义务教育管理体制，将市属初中和部分高中学校下放给区管理。加速发展高中阶段教育，扩大普通高中和职业高中招生规模。加快贵阳学院基础设施和学科专业建设，组建贵阳高等职业技术学院和卫生护理学院。优化教育结构，鼓励社会力量办学。加快发展职业教育、成人教育和远程教育，努力构建全民学习、终身学习的学习型社会。实施人才强市战略。树立人才是第一资源的观念，尊重知识、尊重人才、尊重劳动、尊重创造，促进人才资源和经济社会发展相协调。大力推进电子政务、电子商务建设，加快发展信息家电和网上服务，建立信息化社区。提高中小城镇信息化水平，加速区域信息化进程，把贵阳建设成为全国信息化先进城市。

二是加快发展文化卫生等社会事业。深入挖掘历史文化，大力弘扬革命文化，努力繁荣现代文化，积极推进文化事业和文化产业发展。加强公共卫生建设。进一步健全公共卫生体系，全力做好重大传染病的预防控制和公共卫生突发事件的应急处理,加快乡镇卫生院的改造和设备更新，改善农村医疗条件。深入开展爱国卫生运动。全面发展其它各项社会事业。加强人口资源环境工作，促进人与自然和谐发展。做好基层基础工作，稳定低生育水平，提高出生人口素质。

三是大力推进社会主义精神文明建设。切实把全市人民的思想和行动凝聚到全面建设小康社会的目标上来。以创建文明城市为目标，以社区为基础，继续开展“争做文明市民、营造优美环境、创建文明城市”活动，全面贯彻落实《公民道德建设实施纲要》，加快普及科学知识，倡导科学文明和健康向上的生活方式，提高城乡居民素质和社会文明程度。

（八）切实做好关系人民群众利益的各项工作，努力构建和谐社会

一是大力促进就业和再就业。把就业和再就业工作作为经济工作的重要组成部分和重要考核指标来组织和安排，列入国民经济和社会发展的规划和计划中，加大落实和检查力度，更好地发挥在构建和谐社会中的核心作用。大力发展劳动密集型、劳动服务型的中小企业，力争为社会提供更多的就业岗位。创新就业方式和门路，通过发展个体经济，培育临时就业等措施，鼓励人们创业发展。努力做好下岗失业人员的再就业工作，抓好培训、联系、服务等重要环节，不断提高就业服务水平。切实落实促进就业再就业的一系列政策，加大劳动力市场的建设培育力度，形成促进就业再就业的良性体制和机制。

二是做好关系群众利益的各项工作，努力提高人民生活水平。进一步完善社会保障体系建设。继续完善社会统筹和个人账户相结合的养老、医疗、失业保险和社会救济制度，健全社会医疗救助和多层次的医疗保障体系，实现国有下岗职工基本生活保障与失业保险并轨。依法扩大养老、医疗、失业、工伤保险覆盖面，启动生育保险，重点抓好非公有制企业和灵活就业人员的参保工作，切实做到应保尽保，足额发放。完善城镇居民最低生活保障制度，合理确定保障标准和方式，并逐步向农村覆盖，不断提高保障水平。探索建立农村特困群众医疗救助制度，解决农村贫困群众的基本生活。进一步完善农村困难群众的救助制度，尽可能地把农村居民最低生活保障制度从目前的三个区扩展到有条件的区市县。同时，积极探索失地农民的就业、生产、生活的保障问题。加大经济适用住房和廉租房的建设力度，真正实现居者有其屋的目标。

三是全力维护社会稳定。坚持“一把手”负总责，落实社会治安综合治理领导责任制、安全生产责任制和信访工作责任制，层层落实责任，形成党政统一领导、部门齐抓共管、社会整体联动的工作格局。坚持预防为主，把不稳定因素消灭在萌芽状态。充分发挥乡村、街委、企业的作用，健全制度，完善网络，把不稳定因素控制在基层。对一些老大难的信访案件，将落实包案领导和责任单位，限期解决。对企业改制、土地征用、移民搬迁、拆迁安置、拖欠农民工工资等引发的矛盾纠纷进行全面梳理，切实做好工作。妥善处理各种案件，构筑维护稳定的防线。建立和完善突发事件的预警机制。一旦发生群体性、突发性事件，所在地区、部门和单位领导要亲临一线，缓解矛盾，控制事态，妥善处理。加强安全生产管理。严格执行安全生产责任制和责任追究制，加大专项整治力度，依法取缔、关闭证照不全或不具备安全生产条件的煤矿、矿山。同时，切实抓好交通运输、食品卫生、消防等方面的安全工作，坚决防止和遏制重特大事故的发生。落实“一岗双责”制，切实加强公安队伍建设，强化社会治安防控体系建设，推进社会治安综合治理，努力打造“平安贵阳”。进一步加强流动人口管理，重点打击严重暴力犯罪、黑恶势力、有组织犯罪及毒品犯罪，把打击 “两抢一盗”和净化校园周边治安环境等侵财性犯罪作为重中之重，增强群众的安全感，保障人民群众安居乐业。

（九）切实转变政府职能，努力提高工作效能

一是依法界定政府职能。对照行政许可法，继续清理行政审批项目，坚决纠正不符合要求的行政许可，制定严密的后续监管措施，推行严格的过错追究制。对必须行使的行政审批事项，积极推行统一办理、联合办理、集中办理，努力减少中间环节。加快建立健全各种应急机制，提高政府应对突发事件和公共危机的能力。认真履行政府的公共服务功能，把财力物力等公共资源更多地放在社会发展和解决民生问题上，使政府在职能转变中，有所为有所不为，做到不该干的事坚决不干，应该干的事坚决干好。

二是推进政府管理方式创新。以全面推行政务公开为切入点，有计划分步骤地构建多方式和多渠道的政务公开平台，及时便捷地把政府决策程序、服务方式、办事途径向社会公布，扩大公众参与权、知情权，自觉接受人大的法律监督、政协的民主监督和人民群众的监督。进一步扩大政务公开的内容和范围，拓展政策、信息公开的渠道和载体，强化政策的监督实施和效果评价。在完善市级政务公开的基础上，重点推进乡镇和县级政务公开的制度化、规范化，增强行政权力运行的透明度，防止权力的滥用。推进决策科学化、民主化，推进办事程序规范化、制度化，坚决杜绝乱打招呼、乱批条子，不按规章制度办事的行为。严肃各项纪律，坚决制止有令不行、有禁不止的行为。按照运转高效的要求，健全管理制度，切实加快工作节奏、提高办事效率。从实际出发，探索政府服务企业的新方式，建立完善政府联系重点企业制度，主动帮助企业解决发展中遇到的问题。

三是强化行政权力监督。有权必有责，用权受监督，侵权要赔偿。坚持权责一致的原则，按照法定的权限和程序履行职责，明确违法行政必须承担的法律责任，慎对权力，慎用权力。加大责任追究力度，完善政府廉政工作制度体系，进一步规范领导干部从政行为。

各位代表，今年是“十五”计划的最后一年，各项工作任务十分繁重，必须付出更大的努力。让我们紧密团结在以胡锦涛同志为总书记的党中央周围，以邓小平理论和“三个代表”重要思想为指导，全面贯彻十六大和十六届三中、四中全会精神，在省委、省政府和市委的领导下，振奋精神，坚定信心，开拓进取，扎实工作，加快发展，为全面完成今年的各项目标任务，实现建设“大贵阳”和“两个率先”的宏伟目标而努力奋斗！

让我们携起手来，共同创造贵阳更加美好的明天！
